# Supplementary material for: Overlapping cell population expression profiling and regulatory inference in C. elegans
Source: BMC Genomics. 2016 Feb 29;17:159. doi: 10.1186/s12864-016-2482-z (PMC4772325; doi:10.1186/s12864-016-2482-z)
Supplement: Additional file 13: — Web supplement. (DOC 21 kb) [file 12864_2016_2482_MOESM13_ESM.zip › sortWeb/clusters/hier.300.clusters/295.html]

Cluster 295 

## Cluster 295

### Expression

| cnd-1 rep. 1 | cnd-1 rep. 2 | cnd-1 rep. 3 | pha-4 rep. 1 | pha-4 rep. 2 | pha-4 rep. 3 | ceh-27 | ceh-36 | ceh-6 | F21D5.9 | mir-57 | mls-2 | pal-1 | pros-1 | ttx-3 | unc-130 | hlh-16 | irx-1 | ceh-6 (+) hlh-16 (+) | ceh-6 (+) hlh-16 (-) | ceh-6 (-) hlh-16 (+) | cnd-1 singlets | pha-4 singlets | 0 | 60 | 120 | 150 | 180 | 240 | 330 | 390 | 420 | 480 | 540 | 570 | 600 | 630 | 660 | NAME | Functional description |
| --- | --- | --- | --- | --- | --- | --- | --- | --- | --- | --- | --- | --- | --- | --- | --- | --- | --- | --- | --- | --- | --- | --- | --- | --- | --- | --- | --- | --- | --- | --- | --- | --- | --- | --- | --- | --- | --- | --- | --- |
|  |  |  |  |  |  |  |  |  |  |  |  |  |  |  |  |  |  |  |  |  |  |  |  |  |  |  |  |  |  |  |  |  |  |  |  |  |  | K05C4.8 |  |
|  |  |  |  |  |  |  |  |  |  |  |  |  |  |  |  |  |  |  |  |  |  |  |  |  |  |  |  |  |  |  |  |  |  |  |  |  |  | C01F1.4 |  |
|  |  |  |  |  |  |  |  |  |  |  |  |  |  |  |  |  |  |  |  |  |  |  |  |  |  |  |  |  |  |  |  |  |  |  |  |  |  | K07D4.9 |  |
|  |  |  |  |  |  |  |  |  |  |  |  |  |  |  |  |  |  |  |  |  |  |  |  |  |  |  |  |  |  |  |  |  |  |  |  |  |  | C47G2.6 |  |
|  |  |  |  |  |  |  |  |  |  |  |  |  |  |  |  |  |  |  |  |  |  |  |  |  |  |  |  |  |  |  |  |  |  |  |  |  |  | *best-22* | BESTrophin (chloride channel) homolog |
|  |  |  |  |  |  |  |  |  |  |  |  |  |  |  |  |  |  |  |  |  |  |  |  |  |  |  |  |  |  |  |  |  |  |  |  |  |  | F36H1.11 |  |
|  |  |  |  |  |  |  |  |  |  |  |  |  |  |  |  |  |  |  |  |  |  |  |  |  |  |  |  |  |  |  |  |  |  |  |  |  |  | *mec-9* | MEChanosensory abnormality |
|  |  |  |  |  |  |  |  |  |  |  |  |  |  |  |  |  |  |  |  |  |  |  |  |  |  |  |  |  |  |  |  |  |  |  |  |  |  | M01A8.2 |  |
|  |  |  |  |  |  |  |  |  |  |  |  |  |  |  |  |  |  |  |  |  |  |  |  |  |  |  |  |  |  |  |  |  |  |  |  |  |  | *lron-14* | eLRR (extracellular Leucine-Rich Repeat) ONly |
|  |  |  |  |  |  |  |  |  |  |  |  |  |  |  |  |  |  |  |  |  |  |  |  |  |  |  |  |  |  |  |  |  |  |  |  |  |  | C23H5.12 |  |
|  |  |  |  |  |  |  |  |  |  |  |  |  |  |  |  |  |  |  |  |  |  |  |  |  |  |  |  |  |  |  |  |  |  |  |  |  |  | Y45F3A.4 |  |
|  |  |  |  |  |  |  |  |  |  |  |  |  |  |  |  |  |  |  |  |  |  |  |  |  |  |  |  |  |  |  |  |  |  |  |  |  |  | ZK899.1 |  |
|  |  |  |  |  |  |  |  |  |  |  |  |  |  |  |  |  |  |  |  |  |  |  |  |  |  |  |  |  |  |  |  |  |  |  |  |  |  | C18D1.2 |  |
|  |  |  |  |  |  |  |  |  |  |  |  |  |  |  |  |  |  |  |  |  |  |  |  |  |  |  |  |  |  |  |  |  |  |  |  |  |  | C54D10.5 |  |
|  |  |  |  |  |  |  |  |  |  |  |  |  |  |  |  |  |  |  |  |  |  |  |  |  |  |  |  |  |  |  |  |  |  |  |  |  |  | M176.5 |  |
|  |  |  |  |  |  |  |  |  |  |  |  |  |  |  |  |  |  |  |  |  |  |  |  |  |  |  |  |  |  |  |  |  |  |  |  |  |  | M60.6 |  |
|  |  |  |  |  |  |  |  |  |  |  |  |  |  |  |  |  |  |  |  |  |  |  |  |  |  |  |  |  |  |  |  |  |  |  |  |  |  | *lec-3* | gaLECtin |
|  |  |  |  |  |  |  |  |  |  |  |  |  |  |  |  |  |  |  |  |  |  |  |  |  |  |  |  |  |  |  |  |  |  |  |  |  |  | F07H5.3 |  |
|  |  |  |  |  |  |  |  |  |  |  |  |  |  |  |  |  |  |  |  |  |  |  |  |  |  |  |  |  |  |  |  |  |  |  |  |  |  | *lin-33* | abnormal cell LINeage |
|  |  |  |  |  |  |  |  |  |  |  |  |  |  |  |  |  |  |  |  |  |  |  |  |  |  |  |  |  |  |  |  |  |  |  |  |  |  | C43H6.3 |  |
|  |  |  |  |  |  |  |  |  |  |  |  |  |  |  |  |  |  |  |  |  |  |  |  |  |  |  |  |  |  |  |  |  |  |  |  |  |  | T04C9.7 |  |
|  |  |  |  |  |  |  |  |  |  |  |  |  |  |  |  |  |  |  |  |  |  |  |  |  |  |  |  |  |  |  |  |  |  |  |  |  |  | F52D2.5 |  |
|  |  |  |  |  |  |  |  |  |  |  |  |  |  |  |  |  |  |  |  |  |  |  |  |  |  |  |  |  |  |  |  |  |  |  |  |  |  | *math-1* | MATH (meprin-associated Traf homology) domain containing |
|  |  |  |  |  |  |  |  |  |  |  |  |  |  |  |  |  |  |  |  |  |  |  |  |  |  |  |  |  |  |  |  |  |  |  |  |  |  | F07C6.3 |  |
|  |  |  |  |  |  |  |  |  |  |  |  |  |  |  |  |  |  |  |  |  |  |  |  |  |  |  |  |  |  |  |  |  |  |  |  |  |  | VW02B12L.2 |  |
|  |  |  |  |  |  |  |  |  |  |  |  |  |  |  |  |  |  |  |  |  |  |  |  |  |  |  |  |  |  |  |  |  |  |  |  |  |  | F21F3.6 |  |
|  |  |  |  |  |  |  |  |  |  |  |  |  |  |  |  |  |  |  |  |  |  |  |  |  |  |  |  |  |  |  |  |  |  |  |  |  |  | R03G5.6 |  |
|  |  |  |  |  |  |  |  |  |  |  |  |  |  |  |  |  |  |  |  |  |  |  |  |  |  |  |  |  |  |  |  |  |  |  |  |  |  | F22D3.6 |  |
|  |  |  |  |  |  |  |  |  |  |  |  |  |  |  |  |  |  |  |  |  |  |  |  |  |  |  |  |  |  |  |  |  |  |  |  |  |  | E02A10.3 |  |
|  |  |  |  |  |  |  |  |  |  |  |  |  |  |  |  |  |  |  |  |  |  |  |  |  |  |  |  |  |  |  |  |  |  |  |  |  |  | *dma-1* | Dendrite Morphology Abnormal |
|  |  |  |  |  |  |  |  |  |  |  |  |  |  |  |  |  |  |  |  |  |  |  |  |  |  |  |  |  |  |  |  |  |  |  |  |  |  | B0507.2 |  |
|  |  |  |  |  |  |  |  |  |  |  |  |  |  |  |  |  |  |  |  |  |  |  |  |  |  |  |  |  |  |  |  |  |  |  |  |  |  | *frm-1* | FERM domain (protein4.1-ezrin-radixin-moesin) family |
|  |  |  |  |  |  |  |  |  |  |  |  |  |  |  |  |  |  |  |  |  |  |  |  |  |  |  |  |  |  |  |  |  |  |  |  |  |  | *mgl-3* | Metabotropic GLutamate receptor family |
|  |  |  |  |  |  |  |  |  |  |  |  |  |  |  |  |  |  |  |  |  |  |  |  |  |  |  |  |  |  |  |  |  |  |  |  |  |  | F47A4.5 |  |
|  |  |  |  |  |  |  |  |  |  |  |  |  |  |  |  |  |  |  |  |  |  |  |  |  |  |  |  |  |  |  |  |  |  |  |  |  |  | C03A3.2 |  |
|  |  |  |  |  |  |  |  |  |  |  |  |  |  |  |  |  |  |  |  |  |  |  |  |  |  |  |  |  |  |  |  |  |  |  |  |  |  | *drh-2* | Dicer Related Helicase |
|  |  |  |  |  |  |  |  |  |  |  |  |  |  |  |  |  |  |  |  |  |  |  |  |  |  |  |  |  |  |  |  |  |  |  |  |  |  | Y43F8A.5 |  |
|  |  |  |  |  |  |  |  |  |  |  |  |  |  |  |  |  |  |  |  |  |  |  |  |  |  |  |  |  |  |  |  |  |  |  |  |  |  | ZC15.3 |  |
|  |  |  |  |  |  |  |  |  |  |  |  |  |  |  |  |  |  |  |  |  |  |  |  |  |  |  |  |  |  |  |  |  |  |  |  |  |  | Y105E8A.1 |  |
|  |  |  |  |  |  |  |  |  |  |  |  |  |  |  |  |  |  |  |  |  |  |  |  |  |  |  |  |  |  |  |  |  |  |  |  |  |  | C30E1.9 |  |
|  |  |  |  |  |  |  |  |  |  |  |  |  |  |  |  |  |  |  |  |  |  |  |  |  |  |  |  |  |  |  |  |  |  |  |  |  |  | Y44A6D.2 |  |
|  |  |  |  |  |  |  |  |  |  |  |  |  |  |  |  |  |  |  |  |  |  |  |  |  |  |  |  |  |  |  |  |  |  |  |  |  |  | *pgal-1* | PeptidylGlycine alpha-Amidating Lyase |
|  |  |  |  |  |  |  |  |  |  |  |  |  |  |  |  |  |  |  |  |  |  |  |  |  |  |  |  |  |  |  |  |  |  |  |  |  |  | T01D3.1 |  |
|  |  |  |  |  |  |  |  |  |  |  |  |  |  |  |  |  |  |  |  |  |  |  |  |  |  |  |  |  |  |  |  |  |  |  |  |  |  | C29H12.6 |  |
|  |  |  |  |  |  |  |  |  |  |  |  |  |  |  |  |  |  |  |  |  |  |  |  |  |  |  |  |  |  |  |  |  |  |  |  |  |  | *cmd-1* | CalMoDulin |
|  |  |  |  |  |  |  |  |  |  |  |  |  |  |  |  |  |  |  |  |  |  |  |  |  |  |  |  |  |  |  |  |  |  |  |  |  |  | F33G12.6 |  |
|  |  |  |  |  |  |  |  |  |  |  |  |  |  |  |  |  |  |  |  |  |  |  |  |  |  |  |  |  |  |  |  |  |  |  |  |  |  | *zfh-2* | Zinc Finger and Homeobox |
|  |  |  |  |  |  |  |  |  |  |  |  |  |  |  |  |  |  |  |  |  |  |  |  |  |  |  |  |  |  |  |  |  |  |  |  |  |  | *lgc-40* | Ligand-Gated ion Channel |
|  |  |  |  |  |  |  |  |  |  |  |  |  |  |  |  |  |  |  |  |  |  |  |  |  |  |  |  |  |  |  |  |  |  |  |  |  |  | *nlp-18* | Neuropeptide-Like Protein |
|  |  |  |  |  |  |  |  |  |  |  |  |  |  |  |  |  |  |  |  |  |  |  |  |  |  |  |  |  |  |  |  |  |  |  |  |  |  | T25B9.10 |  |
|  |  |  |  |  |  |  |  |  |  |  |  |  |  |  |  |  |  |  |  |  |  |  |  |  |  |  |  |  |  |  |  |  |  |  |  |  |  | *flp-27* | FMRF-Like Peptide |
|  |  |  |  |  |  |  |  |  |  |  |  |  |  |  |  |  |  |  |  |  |  |  |  |  |  |  |  |  |  |  |  |  |  |  |  |  |  | *lfi-1* | Lin-5 (Five) Interacting protein |
|  |  |  |  |  |  |  |  |  |  |  |  |  |  |  |  |  |  |  |  |  |  |  |  |  |  |  |  |  |  |  |  |  |  |  |  |  |  | *cutl-15* | CUTiclin-Like |
|  |  |  |  |  |  |  |  |  |  |  |  |  |  |  |  |  |  |  |  |  |  |  |  |  |  |  |  |  |  |  |  |  |  |  |  |  |  | *snt-3* | SyNapTotagmin |
|  |  |  |  |  |  |  |  |  |  |  |  |  |  |  |  |  |  |  |  |  |  |  |  |  |  |  |  |  |  |  |  |  |  |  |  |  |  | *linc-128* | Long Intervening Non-Coding RNA |
|  |  |  |  |  |  |  |  |  |  |  |  |  |  |  |  |  |  |  |  |  |  |  |  |  |  |  |  |  |  |  |  |  |  |  |  |  |  | C18F10.7 |  |
|  |  |  |  |  |  |  |  |  |  |  |  |  |  |  |  |  |  |  |  |  |  |  |  |  |  |  |  |  |  |  |  |  |  |  |  |  |  | T04C9.1 |  |
|  |  |  |  |  |  |  |  |  |  |  |  |  |  |  |  |  |  |  |  |  |  |  |  |  |  |  |  |  |  |  |  |  |  |  |  |  |  | *add-1* | ADDucin |

### Phenotypes enriched

none found

### Anatomy terms enriched

none found

### GO terms enriched

none found

### Expression clusters enriched

|  |  |  |  |
| --- | --- | --- | --- |
| **Group name** | **Number in cluster** | **Enrichment** | **FDR corrected p** |
| Larval Pan-neural Enriched Genes. | 22 | 3.85 | 5.67e-06 |
| Genes enriched in neuronal miRNA-induced silencing complexes (miRISC) as detected by immunoprecipitations and microarray analysis. | 15 | 5.86 | 1.05e-05 |
| WT-Pico Pan-neural Enriched Genes, with genes found multiple times in a single dataset removed (without dups). | 21 | 3.54 | 5.08e-05 |
| Genes significantly enriched (> 2x, FDR < 5%) in a particular cell-type versus a reference sample of all cells at the same stage. WBPaper00037950:A-class-motor-neurons\_larva\_enriched | 12 | 4.71 | 2.11e-03 |
| Genes significantly enriched (> 2x, FDR < 5%) in a particular cell-type versus a reference sample of all cells at the same stage. WBPaper00037950:all-neurons\_larva\_enriched | 14 | 3.74 | 4.21e-03 |
| Larval A-class motor neuron enriched genes. | 8 | 5.35 | 2.65e-02 |
| Genes with significant variation across both genotypes and environments. Genotypes | 14 | 3.09 | 2.69e-02 |
| Genes significantly enriched (> 2x, FDR < 5%) in a particular cell-type versus a reference sample of all cells at the same stage. WBPaper00037950:glr-1(+)-neurons\_larva\_enriched | 10 | 4.09 | 3.19e-02 |

### Motifs enriched

|  |  |  |  |  |  |
| --- | --- | --- | --- | --- | --- |
| **Motif** | **Logo** | **Possible orthologs** | **Number of motifs in cluster** | **Enrichment** | **FDR corrected p** |
| ALX3\_3 |  | alr-1 cfi-1 ZC204.2 | 24 | 4.26 | 1.8e-07 |
| pTH10722 |  | eor-1 ref-2 egrh-3 | 42 | 2.27 | 2.4e-07 |
| SRP000217\_Klf4 |  | klf-2 klf-1 | 42 | 2.15 | 1.2e-06 |
| MA0452.2 |  | B0310.2 | 44 | 2.02 | 1.9e-06 |
| V$NKX25\_02 |  | ceh-24 (0.7) ceh-31 (0.66) ceh-8 (0.64) ceh-9 (0.56) ceh-30 ceh-19 alr-1 ceh-43 lin-39 lim-7 ceh-1 | 29 | 2.99 | 2.9e-06 |
| MA0474.1 |  | lin-1 C24A1.2 | 55 | 1.53 | 3.3e-06 |
| FOXJ2\_3 |  | fkh-10 (0.58) fkh-8 (0.51) fkh-7 daf-16 pha-4 lin-31 let-381 | 43 | 1.99 | 6.2e-06 |
| MA0037.2 |  | elt-1 ztf-29 | 44 | 1.93 | 7.0e-06 |
| MA0079.3 |  | klf-2 ZC328.2 klf-1 | 33 | 2.52 | 7.9e-06 |
| Antp\_FlyReg\_FBgn0000095 |  | hmg-12 ceh-43 lin-39 lin-31 ceh-53 let-381 Y116A8C.22 | 31 | 2.65 | 9.3e-06 |
| MA0472.1 |  | ZC328.2 daf-16 | 44 | 1.90 | 1.2e-05 |
| GM12878\_EGR1\_HudsonAlpha |  | klf-2 ZC328.2 klf-1 | 36 | 2.28 | 1.2e-05 |
| MA0446.1 |  | lin-31 let-381 | 37 | 2.21 | 1.4e-05 |
| Hth\_SOLEXA\_FBgn0001235 |  | ces-1 (-0.64) ceh-20 ceh-32 F55C5.11 | 38 | 2.15 | 1.6e-05 |
| Hoxc13\_3127 |  | ceh-24 (0.7) pal-1 (-0.56) | 28 | 2.83 | 1.7e-05 |
| V$POU3F2\_02 |  | ceh-18 (0.8) sox-4 (0.68) tbp-1 | 31 | 2.58 | 1.7e-05 |
| ftz\_FlyReg\_FBgn0001077 |  | ceh-31 (0.66) ceh-16 (-0.56) ceh-9 (0.56) alr-1 ceh-43 lin-39 lim-7 ceh-1 | 38 | 2.15 | 1.7e-05 |
| MA0163.1 |  | Y53H1A.2 | 35 | 2.29 | 2.0e-05 |
| V$POU3F2\_01 |  | ceh-18 (0.8) dmd-3 (-0.56) | 34 | 2.34 | 2.3e-05 |
| TBX3\_f1 |  | ceh-45 tbx-39 | 39 | 2.07 | 2.4e-05 |
| Klf7\_0974 |  | klf-2 klf-1 sptf-3 | 37 | 2.13 | 3.5e-05 |
| POU4F2\_1 |  | ceh-18 (0.8) unc-86 | 32 | 2.41 | 3.6e-05 |
| MA0543.1 |  | eor-1 daf-8 | 38 | 2.07 | 4.1e-05 |
| pTH9082 |  | mab-23 | 26 | 2.85 | 5.2e-05 |
| MA0451.1 |  | nhr-2 (-0.53) php-3 | 29 | 2.58 | 5.5e-05 |
| sqz\_SANGER\_5\_FBgn0010768 |  | mel-28 (-0.53) fkh-7 lin-29 | 43 | 1.84 | 5.9e-05 |
| Cdx2\_4272 |  | ceh-13 | 39 | 2.00 | 6.1e-05 |
| SMAD3\_1 |  | daf-8 | 50 | 1.59 | 6.5e-05 |
| Hoxb8\_3780 |  | lin-39 | 28 | 2.63 | 6.5e-05 |
| Mw137 |  | blmp-1 | 36 | 2.12 | 6.8e-05 |
| N$DAF16\_01 |  | fkh-10 (0.58) fkh-8 (0.51) fkh-7 daf-16 lin-31 let-381 | 35 | 2.17 | 6.9e-05 |
| MA0033.1 |  | hlh-8 hlh-15 lin-31 hlh-32 ngn-1 | 20 | 3.54 | 8.2e-05 |
| Repo\_Cell\_FBgn0011701 |  | ceh-18 (0.8) ceh-31 (0.66) ceh-16 (-0.56) pal-1 (-0.56) ceh-23 (0.51) lim-4 ceh-30 ceh-12 pha-2 ceh-36 alr-1 ceh-14 ceh-43 cog-1 lin-39 lim-7 lim-6 ceh-45 ceh-10 ceh-53 and 6 others  [full list] | 30 | 2.45 | 8.4e-05 |
| TBX2\_1 |  | tbx-38 mab-9 tbx-39 tbx-43 | 51 | 1.55 | 9.4e-05 |
| Tbp\_pr781 |  | tbp-1 | 42 | 1.84 | 9.5e-05 |
| Egr1\_2580 |  | ZC328.2 | 52 | 1.51 | 9.8e-05 |
| pTH6641 |  | lin-31 | 48 | 1.64 | 1.0e-04 |
| K562\_ZBTB7A\_HudsonAlpha |  | ZC328.2 | 34 | 2.17 | 1.2e-04 |
| pTH6447 |  | ceh-19 | 35 | 2.11 | 1.4e-04 |
| Hoxc8\_3429 |  | lin-39 | 35 | 2.11 | 1.4e-04 |
| NR1D1\_f1 |  | nhr-213 nhr-118 | 32 | 2.26 | 1.4e-04 |
| NR2F6\_f1 |  | nhr-2 (-0.53) nhr-239 | 48 | 1.62 | 1.5e-04 |
| pTH6108 |  | fkh-10 (0.58) lin-31 let-381 C34D1.1 | 48 | 1.62 | 1.5e-04 |
| V$MEF2\_04 |  | mef-2 | 37 | 1.99 | 1.8e-04 |
| Dlx4\_3488 |  | ceh-12 alr-1 npax-3 ceh-43 lin-39 ceh-45 ceh-53 ceh-1 | 11 | 6.57 | 1.9e-04 |
| Hoxd13\_2356 |  | pal-1 (-0.56) | 41 | 1.83 | 2.0e-04 |
| pTH9957 |  | irx-1 (-0.51) daf-16 fkh-9 | 43 | 1.76 | 2.0e-04 |
| V$FAC1\_01 |  | gei-8 | 37 | 1.98 | 2.1e-04 |
| nau\_SANGER\_5\_FBgn0002922 |  | hlh-1 hlh-15 hlh-14 hlh-11 | 55 | 1.39 | 2.1e-04 |
| Pax7\_3783 |  | alr-1 lim-7 | 35 | 2.06 | 2.2e-04 |
| Hnf4a\_2640 |  | nhr-62 | 33 | 2.16 | 2.3e-04 |
| Rax\_3443 |  | alr-1 lin-39 eyg-1 ceh-45 ceh-10 ceh-1 | 25 | 2.69 | 2.4e-04 |
| rn\_SOLEXA\_5\_FBgn0259172 |  | lin-29 | 19 | 3.43 | 2.4e-04 |
| pTH9137 |  | nhr-65 | 26 | 2.58 | 2.8e-04 |
| ZN384\_f1 |  | lin-29 K11D2.4 | 26 | 2.58 | 2.8e-04 |
| Cdx1\_2245 |  | ceh-13 | 37 | 1.95 | 3.0e-04 |
| pTH5778 |  | egl-5 | 28 | 2.42 | 3.1e-04 |
| pTH6591 |  | lin-31 | 38 | 1.91 | 3.1e-04 |
| HLF\_1 |  | ces-2 (-0.74) atf-2 C48E7.11 Y51H4A.4 F23F12.9 | 21 | 3.06 | 3.3e-04 |
| Srf\_3509 |  | unc-120 | 11 | 6.14 | 3.4e-04 |
| Six4\_2860 |  | ceh-32 | 48 | 1.57 | 3.5e-04 |
| Hoxc10\_2779 |  | php-3 lin-39 lim-7 | 29 | 2.32 | 3.8e-04 |
| pTH10795 |  | ZC328.2 ztf-14 lin-1 ref-2 | 33 | 2.10 | 3.8e-04 |
| Hoxd10\_2368 |  | php-3 lin-39 | 26 | 2.51 | 4.3e-04 |
| V$PAX6\_01 |  | pax-3 pax-2 | 16 | 3.85 | 4.5e-04 |
| HXA10\_f1 |  | php-3 lin-39 hbl-1 | 30 | 2.22 | 5.5e-04 |
| ONEC2\_si |  | ceh-48 | 32 | 2.11 | 5.5e-04 |
| pTH10623 |  | scrt-1 | 27 | 2.40 | 5.6e-04 |
| Dll\_Cell\_FBgn0000157 |  | pal-1 (-0.56) ceh-43 lin-39 | 21 | 2.93 | 5.8e-04 |
| MA0535.1 |  | daf-8 pax-2 | 14 | 4.31 | 5.9e-04 |
| pTH10772 |  | ceh-52 | 22 | 2.81 | 6.0e-04 |
| HLH4C\_da\_SANGER\_5\_4\_FBgn0011277 |  | ces-1 (-0.64) hlh-2 (-0.62) hlh-1 hlh-15 | 38 | 1.85 | 6.0e-04 |
| pTH9296 |  | ztf-6 (-0.51) C34D1.1 gei-11 | 42 | 1.72 | 6.2e-04 |
| Hoxa7\_2668 |  | lin-39 | 26 | 2.45 | 6.4e-04 |
| HOXA13\_2 |  | ceh-24 (0.7) pal-1 (-0.56) php-3 ceh-13 lin-39 T27F2.4 | 35 | 1.96 | 6.4e-04 |
| MA0538.1 |  | ceh-9 (0.56) hif-1 ztf-3 daf-12 Y5F2A.4 | 29 | 2.24 | 6.9e-04 |
| pTH8985 |  | athp-1 | 25 | 2.51 | 7.1e-04 |
| Barx2\_3447 |  | ceh-43 | 33 | 2.04 | 7.1e-04 |
| SOX2\_4 |  | sox-4 (0.68) dmd-4 grh-1 | 46 | 1.59 | 7.1e-04 |
| pTH10647 |  | nhr-232 | 41 | 1.74 | 7.1e-04 |
| HUVEC\_GATA2\_UCD |  | elt-1 | 38 | 1.84 | 7.3e-04 |
| ETS2\_f1 |  | lin-1 C24A1.2 | 42 | 1.70 | 7.6e-04 |
| MA0118.1 |  | ref-2 | 33 | 2.03 | 7.8e-04 |
| MA0048.1 |  | hlh-1 hlh-15 | 51 | 1.46 | 7.8e-04 |
| Hoxa7\_3750 |  | lin-39 | 33 | 2.02 | 8.2e-04 |
| pTH9222 |  | mel-28 (-0.53) | 36 | 1.90 | 8.2e-04 |
| Hoxa10\_2318 |  | ceh-24 (0.7) | 27 | 2.34 | 8.2e-04 |
| Hoxa11\_2218 |  | php-3 | 16 | 3.64 | 8.3e-04 |
| pTH10027 |  | M03D4.4 | 19 | 3.10 | 8.7e-04 |
| MA0062.2 |  | lin-1 C24A1.2 | 40 | 1.75 | 9.2e-04 |
| HOXA10\_2 |  | ceh-24 (0.7) pal-1 (-0.56) php-3 lin-39 | 21 | 2.83 | 9.3e-04 |
| V$CEBP\_01 |  | C48E7.11 | 29 | 2.20 | 9.9e-04 |
| Hoxd11\_3873 |  | php-3 ceh-13 | 27 | 2.31 | 1.0e-03 |
| Sox17\_2837 |  | sox-4 (0.68) | 47 | 1.55 | 1.0e-03 |
| RORA\_1 |  | nhr-71 (0.53) nhr-2 (-0.53) nhr-68 nhr-6 nhr-213 nhr-10 Y67D8A.3 | 38 | 1.81 | 1.0e-03 |
| HXA9\_f1 |  | ceh-32 lin-39 | 32 | 2.04 | 1.1e-03 |
| pTH9244 |  | tbx-39 | 29 | 2.19 | 1.1e-03 |
| Hoxd8\_2644 |  | ceh-18 (0.8) lin-39 ceh-6 | 24 | 2.52 | 1.1e-03 |
| FOXI1\_f1 |  | lin-31 dro-1 | 25 | 2.44 | 1.1e-03 |
| En1\_3123 |  | ceh-16 (-0.56) ceh-53 | 20 | 2.91 | 1.1e-03 |
| MA0173.1 |  | irx-1 (-0.51) D1081.8 hlh-32 | 39 | 1.77 | 1.1e-03 |
| Plagl1\_0972 |  | Y53H1A.2 | 43 | 1.65 | 1.2e-03 |
| MA0015.1 |  | K11D2.4 che-1 tbp-1 | 19 | 3.02 | 1.2e-03 |
| Sox4 |  | pop-1 (-0.74) nhr-100 (0.54) | 35 | 1.90 | 1.2e-03 |
| pTH6449 |  | ceh-43 | 31 | 2.07 | 1.3e-03 |
| pTH5462 |  | jun-1 (0.64) fos-1 crh-1 F45H11.6 | 40 | 1.73 | 1.3e-03 |
| pTH2846 |  | lin-31 | 46 | 1.56 | 1.3e-03 |
| CUX1\_2 |  | ceh-48 | 38 | 1.79 | 1.4e-03 |
| TBX2\_f1 |  | tbx-39 | 48 | 1.50 | 1.4e-03 |
| FLI1\_f1 |  | lin-1 | 33 | 1.97 | 1.4e-03 |
| PLAG1\_si |  | Y53H1A.2 klf-2 plp-2 | 33 | 1.96 | 1.4e-03 |
| Hoxa6\_1040 |  | lin-39 | 26 | 2.32 | 1.4e-03 |
| pTH9182 |  | tbx-39 | 32 | 2.01 | 1.5e-03 |
| tgo\_sim\_SANGER\_5\_FBgn0015014 |  | mdl-1 (0.76) aha-1 hlh-30 | 33 | 1.96 | 1.5e-03 |
| HMGA1\_f1 |  | mel-28 (-0.53) let-381 Y116A8C.22 Y61A9LA.9 | 35 | 1.88 | 1.5e-03 |
| pTH5511 |  | pha-2 ceh-36 alr-1 ceh-45 ceh-53 | 30 | 2.09 | 1.5e-03 |
| IRX5\_1 |  | irx-1 (-0.51) | 37 | 1.81 | 1.5e-03 |
| pTH3477 |  | daf-16 | 46 | 1.55 | 1.5e-03 |
| V$XFD3\_01 |  | ceh-20 cfi-1 let-381 | 47 | 1.52 | 1.7e-03 |
| pTH8556 |  | pax-2 | 31 | 2.03 | 1.7e-03 |
| Dlx2\_2273 |  | ceh-43 | 21 | 2.70 | 1.7e-03 |
| Hmx3\_3490 |  | ceh-9 (0.56) | 44 | 1.59 | 1.8e-03 |
| pTH5260 |  | lin-22 aha-1 hlh-27 hlh-28 | 28 | 2.17 | 1.8e-03 |
| CREM\_f1 |  | crh-1 ceh-26 | 38 | 1.76 | 1.8e-03 |
| NR4A2\_1 |  | nhr-6 nhr-43 | 25 | 2.35 | 1.8e-03 |
| pTH3469 |  | nhr-2 (-0.53) nhr-213 nhr-19 nhr-10 | 49 | 1.47 | 1.9e-03 |
| Dlx3\_1030 |  | ceh-43 | 26 | 2.29 | 1.9e-03 |
| I$MTTFA\_01 |  | hmg-5 (-0.64) | 32 | 1.97 | 1.9e-03 |
| Vsx1\_1728 |  | alr-1 | 32 | 1.97 | 2.0e-03 |
| Dlx1\_1741 |  | ceh-43 | 12 | 4.42 | 2.1e-03 |
| pTH2283 |  | odd-2 | 24 | 2.40 | 2.1e-03 |
| pTH9215 |  | C34D1.1 | 27 | 2.21 | 2.1e-03 |
| MA0536.1 |  | elt-1 lin-39 | 38 | 1.75 | 2.1e-03 |
| pTH9242 |  | mel-28 (-0.53) | 45 | 1.56 | 2.2e-03 |
| SRF\_2 |  | unc-120 | 25 | 2.32 | 2.3e-03 |
| pTH10837 |  | ceh-24 (0.7) ces-1 (-0.64) T22H9.4 C34H4.5 | 20 | 2.73 | 2.4e-03 |
| MA0026.1 |  | lin-1 C24A1.2 | 37 | 1.77 | 2.4e-03 |
| pTH10718 |  | egl-43 | 38 | 1.74 | 2.4e-03 |
| gl\_SANGER\_5\_FBgn0004618 |  | ces-1 (-0.64) ztf-28 che-1 | 34 | 1.87 | 2.5e-03 |
| MA0531.1 |  | F58G1.2 (-0.53) Y5F2A.4 | 32 | 1.95 | 2.5e-03 |
| pTH5250 |  | C48E7.11 | 24 | 2.37 | 2.5e-03 |
| Hlxb9\_3422 |  | ceh-12 | 22 | 2.53 | 2.5e-03 |
| Elf3\_3876 |  | C24A1.2 | 44 | 1.57 | 2.6e-03 |
| N$SKN1\_01 |  | skn-1 | 45 | 1.55 | 2.6e-03 |
| MA0483.1 |  | odd-1 | 42 | 1.62 | 2.6e-03 |
| Arid3a\_3875 |  | cfi-1 | 44 | 1.57 | 2.6e-03 |
| Pou2f3\_3986 |  | ceh-18 (0.8) | 34 | 1.87 | 2.6e-03 |
| pTH10037 |  | T22C8.4 (-0.53) ref-2 | 25 | 2.30 | 2.6e-03 |
| pTH5914 |  | attf-1 | 28 | 2.12 | 2.7e-03 |
| Lhx1\_2240 |  | lim-7 | 33 | 1.90 | 2.7e-03 |
| V$TATA\_C |  | tbp-1 | 42 | 1.62 | 2.7e-03 |
| pTH4337 |  | crh-1 attf-1 | 23 | 2.42 | 2.8e-03 |
| Hoxb5\_3122 |  | lin-39 | 30 | 2.02 | 2.9e-03 |
| V$NCX\_01 |  | ceh-19 | 32 | 1.93 | 2.9e-03 |
| twi\_da\_SANGER\_5\_FBgn0003900 |  | hlh-8 hlh-32 ngn-1 hlh-12 | 30 | 2.01 | 3.0e-03 |
| pTH9097 |  | Y116A8C.22 | 22 | 2.49 | 3.1e-03 |
| NR2E3\_f1 |  | nhr-100 (0.54) lin-39 lin-1 | 45 | 1.53 | 3.2e-03 |
| MA0503.1 |  | ceh-24 (0.7) pzf-1 | 22 | 2.48 | 3.3e-03 |
| tin\_FlyReg\_FBgn0004110 |  | ceh-22 dsc-1 | 36 | 1.78 | 3.3e-03 |
| Barx1\_2877 |  | ceh-43 | 30 | 1.99 | 3.5e-03 |
| pTH10041 |  | ztf-29 | 23 | 2.39 | 3.5e-03 |
| V$IK2\_01 |  | F26F4.8 (-0.6) | 20 | 2.64 | 3.5e-03 |
| V$BRN2\_01 |  | ceh-18 (0.8) | 42 | 1.60 | 3.5e-03 |
| pTH9237 |  | mel-28 (-0.53) | 22 | 2.46 | 3.6e-03 |
| pTH9256 |  | ceh-18 (0.8) | 27 | 2.13 | 3.6e-03 |
| MA0542.1 |  | ceh-34 elt-3 elt-1 | 43 | 1.57 | 3.6e-03 |
| pTH5267 |  | hlh-32 | 14 | 3.54 | 3.7e-03 |
| pTH5257 |  | C48E7.11 | 33 | 1.87 | 3.7e-03 |
| Tcf7\_0950 |  | pop-1 (-0.74) | 31 | 1.94 | 3.9e-03 |
| pTH10633 |  | R07H5.10 | 43 | 1.57 | 4.0e-03 |
| pTH9951 |  | pal-1 (-0.56) mex-6 | 32 | 1.89 | 4.1e-03 |
| HIF1A\_si |  | hif-1 | 29 | 2.02 | 4.1e-03 |
| ZNF75A\_1 |  | F26F4.8 (-0.6) ztf-3 lag-1 | 33 | 1.85 | 4.2e-03 |
| pTH5877 |  | nhr-100 (0.54) elt-1 | 36 | 1.76 | 4.2e-03 |
| pTH1014 |  | atf-5 | 31 | 1.93 | 4.2e-03 |
| pTH6562 |  | ceh-5 | 34 | 1.82 | 4.2e-03 |
| pTH2684 |  | fos-1 | 52 | 1.36 | 4.3e-03 |
| pTH5159 |  | lin-22 aha-1 mxl-2 mxl-1 C27D6.4 | 29 | 2.01 | 4.3e-03 |
| Sox21\_3417 |  | pop-1 (-0.74) sox-4 (0.68) gei-3 (0.64) C05C9.3 | 19 | 2.69 | 4.4e-03 |
| pTH8863 |  | hmg-12 | 32 | 1.88 | 4.5e-03 |
| CG7386\_F10-12\_SANGER\_5\_FBgn0035691 |  | sox-4 (0.68) F56D1.1 | 23 | 2.34 | 4.5e-03 |
| pTH9297 |  | ceh-18 (0.8) | 27 | 2.10 | 4.6e-03 |
| pTH5924 |  | nhr-255 (0.57) | 23 | 2.34 | 4.6e-03 |
| pTH3998 |  | tbx-39 | 28 | 2.05 | 4.6e-03 |
| pTH10630 |  | lsy-27 | 24 | 2.26 | 4.8e-03 |
| pTH8649 |  | mbr-1 (0.75) | 28 | 2.04 | 4.8e-03 |
| Pou3f1\_3819 |  | ceh-6 | 25 | 2.20 | 4.9e-03 |
| pTH1001 |  | dnj-17 | 33 | 1.84 | 4.9e-03 |
| Elf5 |  | C24A1.2 | 46 | 1.48 | 5.1e-03 |
| pTH1049 |  | elt-1 | 33 | 1.83 | 5.1e-03 |
| FOXK1\_1 |  | nhr-213 lin-31 | 55 | 1.28 | 5.1e-03 |
| pTH6497 |  | lin-31 | 31 | 1.91 | 5.2e-03 |
| pTH3751 |  | tbx-39 | 28 | 2.02 | 5.5e-03 |
| pTH5078 |  | ces-2 (-0.74) | 32 | 1.86 | 5.6e-03 |
| V$TAXCREB\_02 |  | zip-3 crh-1 | 33 | 1.82 | 5.6e-03 |
| Fli1 |  | F19F10.1 lin-1 | 39 | 1.64 | 5.9e-03 |
| V$OCT1\_06 |  | ceh-18 (0.8) | 41 | 1.59 | 6.0e-03 |
| V$PBX1\_01 |  | ceh-20 lin-39 | 33 | 1.81 | 6.2e-03 |
| pTH9125 |  | sox-4 (0.68) egl-13 (0.54) K11D2.4 | 28 | 2.01 | 6.3e-03 |
| pTH5976 |  | irx-1 (-0.51) mxl-1 | 25 | 2.16 | 6.3e-03 |
| pTH8991 |  | cey-3 | 34 | 1.78 | 6.5e-03 |
| pTH9323 |  | odr-7 | 50 | 1.38 | 6.5e-03 |
| Bbx\_3753 |  | gei-3 (0.64) lim-6 cfi-1 nhr-208 | 8 | 5.97 | 6.5e-03 |
| HeLa-S3\_ZNF274\_UCD |  | C28G1.4 | 15 | 3.10 | 7.0e-03 |
| ZBTB49\_1 |  | C46E10.9 aha-1 | 38 | 1.65 | 7.0e-03 |
| pTH9049 |  | ztf-2 | 36 | 1.70 | 7.4e-03 |
| pTH9177 |  | F10B5.3 (0.62) hsf-1 Y53C10A.3 | 42 | 1.55 | 7.5e-03 |
| pTH4325 |  | ceh-18 (0.8) | 26 | 2.07 | 7.8e-03 |
| pTH9220 |  | mbr-1 (0.75) | 28 | 1.98 | 7.8e-03 |
| V$S8\_01 |  | ceh-45 | 26 | 2.07 | 7.9e-03 |
| NR2F1\_3 |  | nhr-2 (-0.53) nhr-213 nhr-15 | 10 | 4.46 | 7.9e-03 |
| CG31670\_SANGER\_5\_FBgn0031375 |  | F21A9.2 CELE\_Y38H8A.5 | 34 | 1.75 | 8.0e-03 |
| pTH3046 |  | Y116A8C.22 | 43 | 1.52 | 8.3e-03 |
| Elf3 |  | C24A1.2 | 42 | 1.54 | 8.5e-03 |
| V$FREAC7\_01 |  | lin-31 | 34 | 1.75 | 8.5e-03 |
| CXXC1\_si |  | F52B11.1 F21D5.4 | 54 | 1.29 | 8.5e-03 |
| pTH9393 |  | ZC416.1 | 29 | 1.92 | 8.6e-03 |
| pTH10030 |  | xbp-1 (0.68) | 28 | 1.96 | 8.6e-03 |
| pTH5887 |  | lin-39 | 34 | 1.75 | 8.6e-03 |
| NR2F1\_2 |  | nhr-2 (-0.53) nhr-19 nhr-62 | 33 | 1.78 | 8.7e-03 |
| pTH5561 |  | nhr-239 | 33 | 1.78 | 8.7e-03 |
| T-47D\_GATA3\_HudsonAlpha |  | elt-1 | 13 | 3.41 | 8.8e-03 |
| Atf1\_3026 |  | crh-1 | 36 | 1.69 | 8.9e-03 |
| Sox1\_2631 |  | sox-4 (0.68) | 24 | 2.15 | 9.2e-03 |
| MA0007.2 |  | nhr-255 (0.57) | 36 | 1.68 | 9.3e-03 |
| Sox15\_3457 |  | sox-4 (0.68) | 16 | 2.85 | 9.3e-03 |
| F$MCM1\_01 |  | unc-120 C24A1.2 | 38 | 1.63 | 9.4e-03 |
| pTH9245 |  | ceh-18 (0.8) | 38 | 1.63 | 9.6e-03 |
| EMX1\_2 |  | ceh-2 | 32 | 1.80 | 9.7e-03 |
| CG8765\_SANGER\_5\_FBgn0036900 |  | H20J04.3 | 37 | 1.65 | 9.8e-03 |
| V$CETS1P54\_02 |  | C52B9.2 | 39 | 1.60 | 9.8e-03 |
| Poxm\_SOLEXA\_5\_FBgn0003129 |  | pax-2 | 31 | 1.83 | 9.8e-03 |
| Gata5\_3768 |  | elt-1 | 33 | 1.76 | 1.0e-02 |
| Mf28 |  | elt-1 | 38 | 1.62 | 1.0e-02 |
| pTH6003 |  | nhr-134 | 39 | 1.60 | 1.0e-02 |
| PRRX1\_f1 |  | alr-1 | 29 | 1.90 | 1.0e-02 |
| pTH9026 |  | attf-1 | 26 | 2.03 | 1.0e-02 |
| Etv3 |  | lin-1 | 38 | 1.62 | 1.0e-02 |
| Mw160 |  | nhr-68 | 38 | 1.62 | 1.1e-02 |
| MCR\_f1 |  | nhr-255 (0.57) | 20 | 2.40 | 1.1e-02 |
| GATA4\_1 |  | end-3 elt-1 | 33 | 1.76 | 1.1e-02 |
| tgo\_ss\_SANGER\_5\_FBgn0015014 |  | aha-1 | 32 | 1.79 | 1.1e-02 |
| pTH9261 |  | dmd-3 (-0.56) pax-3 lin-48 | 15 | 2.95 | 1.1e-02 |
| V$CREB\_02 |  | fos-1 crh-1 | 48 | 1.40 | 1.1e-02 |
| pTH10811 |  | nhr-216 nhr-142 | 23 | 2.18 | 1.1e-02 |
| pTH5080 |  | fos-1 crh-1 | 49 | 1.38 | 1.1e-02 |
| pTH9080 |  | mnm-2 | 34 | 1.72 | 1.2e-02 |
| RFX4\_1 |  | daf-19 (0.62) | 29 | 1.88 | 1.2e-02 |
| PDX1\_1 |  | ceh-31 (0.66) lin-39 ceh-1 | 36 | 1.66 | 1.2e-02 |
| pTH10696 |  | Y44A6D.3 | 24 | 2.10 | 1.2e-02 |
| N$SKN1\_02 |  | ztf-28 skn-1 | 12 | 3.49 | 1.2e-02 |
| Nkx1-2\_3214 |  | ceh-30 | 22 | 2.22 | 1.3e-02 |
| pTH3819 |  | ceh-18 (0.8) | 23 | 2.15 | 1.3e-02 |
| pTH5082 |  | atf-2 | 32 | 1.77 | 1.3e-02 |
| pTH10013 |  | nhr-168 | 35 | 1.68 | 1.3e-02 |
| Bsx\_3483 |  | ceh-31 (0.66) | 28 | 1.91 | 1.3e-02 |
| Pou2f1\_3081 |  | ceh-18 (0.8) | 31 | 1.79 | 1.4e-02 |
| pTH5423 |  | klf-2 | 34 | 1.70 | 1.4e-02 |
| HeLa-S3\_RFX5\_Stanford |  | daf-19 (0.62) | 21 | 2.26 | 1.4e-02 |
| Hoxd1\_3448 |  | ceh-12 | 37 | 1.62 | 1.4e-02 |
| pTH9043 |  | sem-2 | 39 | 1.57 | 1.4e-02 |
| pTH8679 |  | pax-2 | 29 | 1.86 | 1.4e-02 |
| V$VMYB\_01 |  | D1081.8 | 24 | 2.07 | 1.5e-02 |
| MA0244.1 |  | C48E7.11 | 33 | 1.72 | 1.5e-02 |
| Hoxb3\_1720 |  | lin-39 | 29 | 1.85 | 1.5e-02 |
| V$TCF11\_01 |  | skn-1 | 35 | 1.66 | 1.5e-02 |
| pTH6071 |  | C33G8.2 | 32 | 1.74 | 1.6e-02 |
| En2\_0952 |  | ceh-16 (-0.56) | 31 | 1.77 | 1.6e-02 |
| HSFY2\_1 |  | hsf-1 | 36 | 1.63 | 1.6e-02 |
| MA0544.1 |  | gei-11 | 20 | 2.30 | 1.6e-02 |
| Nkx6-1\_2825 |  | cog-1 | 31 | 1.77 | 1.6e-02 |
| MA0161.1 |  | nfi-1 F49E12.6 | 43 | 1.47 | 1.6e-02 |
| Six1\_0935 |  | ceh-32 | 47 | 1.39 | 1.7e-02 |
| CG4854\_SANGER\_10\_FBgn0038766 |  | K11D2.4 | 27 | 1.90 | 1.8e-02 |
| pTH9198 |  | dmd-3 (-0.56) | 33 | 1.70 | 1.8e-02 |
| pTH2936 |  | nhr-239 | 30 | 1.79 | 1.8e-02 |
| pTH8411 |  | tbx-39 | 32 | 1.73 | 1.8e-02 |
| pTH10805 |  | ztf-16 | 29 | 1.82 | 1.8e-02 |
| Nkx1-1\_3856 |  | ceh-30 | 21 | 2.20 | 1.9e-02 |
| pTH3064 |  | crh-1 | 32 | 1.73 | 1.9e-02 |
| Gmeb1\_1745 |  | attf-1 | 25 | 1.98 | 1.9e-02 |
| pTH3084 |  | C01B12.2 attf-1 | 26 | 1.94 | 1.9e-02 |
| Eip93F\_SANGER\_10\_FBgn0013948 |  | mbr-1 (0.75) F26F4.8 (-0.6) | 18 | 2.42 | 2.0e-02 |
| Jundm2\_0911 |  | fos-1 skn-1 | 30 | 1.78 | 2.0e-02 |
| V$GR\_Q6 |  | nhr-255 (0.57) | 23 | 2.07 | 2.0e-02 |
| pTH10823 |  | B0310.2 | 16 | 2.61 | 2.0e-02 |
| YMR043W\_831 |  | unc-120 | 26 | 1.93 | 2.1e-02 |
| Tcf3\_3787 |  | pop-1 (-0.74) | 38 | 1.56 | 2.2e-02 |
| TCF4\_2 |  | hlh-2 (-0.62) | 33 | 1.67 | 2.3e-02 |
| MA0095.2 |  | lsy-2 | 24 | 1.99 | 2.4e-02 |
| V$MYB\_Q6 |  | D1081.8 | 51 | 1.30 | 2.4e-02 |
| Irx6\_2623 |  | irx-1 (-0.51) | 32 | 1.69 | 2.5e-02 |
| pTH6569 |  | ceh-43 | 31 | 1.72 | 2.6e-02 |
| pTH6482 |  | ceh-19 | 27 | 1.85 | 2.6e-02 |
| V$AREB6\_02 |  | ztf-6 (-0.51) | 33 | 1.66 | 2.7e-02 |
| pTH8982 |  | ceh-48 | 32 | 1.69 | 2.7e-02 |
| pTH8399 |  | lin-54 | 31 | 1.71 | 2.8e-02 |
| Hoxa3\_2783 |  | lin-39 | 21 | 2.12 | 2.8e-02 |
| Lbx2\_3869 |  | mls-2 | 29 | 1.77 | 2.9e-02 |
| Mv75 |  | elt-1 | 27 | 1.83 | 2.9e-02 |
| Nkx6-3\_3446 |  | cog-1 | 30 | 1.73 | 3.0e-02 |
| pTH10638 |  | dmd-3 (-0.56) C34D1.1 | 30 | 1.72 | 3.2e-02 |
| BARHL2\_4 |  | ceh-31 (0.66) | 33 | 1.64 | 3.2e-02 |
| Hoxa2\_3079 |  | lin-39 | 28 | 1.79 | 3.2e-02 |
| ERG\_4 |  | lin-1 | 26 | 1.86 | 3.2e-02 |
| pTH6445 |  | ceh-5 | 27 | 1.81 | 3.3e-02 |
| Pou3f4\_3773 |  | ceh-6 | 31 | 1.68 | 3.5e-02 |
| pTH6636 |  | egl-5 | 19 | 2.19 | 3.6e-02 |
| pTH9934 |  | Y53H1A.2 | 28 | 1.77 | 3.7e-02 |
| pTH9353 |  | ceh-51 | 32 | 1.65 | 3.7e-02 |
| Hmbox1\_2674 |  | hmbx-1 | 30 | 1.70 | 3.8e-02 |
| pTH5781 |  | ceh-32 | 51 | 1.28 | 3.8e-02 |
| pTH9384 |  | cfi-1 | 13 | 2.79 | 3.8e-02 |
| Meox1\_2310 |  | ceh-31 (0.66) | 29 | 1.73 | 3.9e-02 |
| pTH9164 |  | ceh-26 | 42 | 1.43 | 3.9e-02 |
| MA0124.1 |  | ceh-24 (0.7) | 30 | 1.69 | 4.3e-02 |
| pTH10769 |  | Y48G1C.6 | 35 | 1.56 | 4.3e-02 |
| Pou3f3\_3235 |  | ceh-6 | 21 | 2.03 | 4.3e-02 |
| pTH10768 |  | med-2 | 26 | 1.81 | 4.4e-02 |
| pTH9708 |  | ceh-34 | 48 | 1.32 | 4.4e-02 |
| pTH10777 |  | dmd-3 (-0.56) | 20 | 2.08 | 4.4e-02 |
| Hoxa5\_3415 |  | lin-39 | 21 | 2.02 | 4.5e-02 |
| pTH10028 |  | nhr-204 | 54 | 1.22 | 4.5e-02 |
| pTH10798 |  | Y75B8A.6 | 50 | 1.29 | 4.7e-02 |
| pTH10816 |  | dmd-6 | 20 | 2.07 | 4.8e-02 |
| pTH9925 |  | ztf-11 | 31 | 1.64 | 4.8e-02 |
| Pbx1\_3203 |  | ceh-20 | 28 | 1.73 | 4.9e-02 |
| V$E47\_02 |  | hlh-2 (-0.62) | 33 | 1.59 | 5.0e-02 |

### Correlated (and anti-correlated) transcription factors

|  |  |
| --- | --- |
| **Transcription factor** | **Correlation** |
| zfh-2 | 0.91 |
| lfi-1 | 0.89 |
| ceh-88 | 0.85 |
| tag-97 | 0.85 |
| saeg-1 | 0.82 |
| ceh-62 | 0.82 |
| ceh-18 | 0.80 |
| unc-42 | 0.78 |
| ctbp-1 | 0.76 |
| mdl-1 | 0.76 |
| mbr-1 | 0.75 |
| nhr-95 | 0.74 |
| madf-4 | 0.73 |
| mgl-2 | 0.73 |
| aptf-4 | 0.73 |
| nhr-198 | 0.72 |
| ceh-54 | 0.71 |
| dmd-10 | 0.71 |
| T18D3.7 | 0.71 |
| saeg-2 | 0.71 |
| ceh-24 | 0.70 |
| nhr-105 | 0.70 |
| daf-3 | 0.69 |
| sox-4 | 0.68 |
| xbp-1 | 0.68 |
| zim-1 | -0.60 |
| Y82E9BR.1 | -0.62 |
| hlh-2 | -0.62 |
| sup-35 | -0.63 |
| ceh-40 | -0.64 |
| hmg-5 | -0.64 |
| ces-1 | -0.64 |
| ztf-13 | -0.65 |
| nhr-64 | -0.66 |
| snpc-1.2 | -0.66 |
| efl-3 | -0.66 |
| dnj-11 | -0.67 |
| ceh-83 | -0.67 |
| dhhc-6 | -0.68 |
| ubxn-1 | -0.68 |
| Y82E9BR.17 | -0.69 |
| F27D4.4 | -0.69 |
| nhr-256 | -0.71 |
| nhr-13 | -0.72 |
| pop-1 | -0.74 |
| ces-2 | -0.74 |
| duxl-1 | -0.76 |
| zip-7 | -0.77 |
| sex-1 | -0.78 |
| Y53F4B.3 | -0.79 |

### ChIP peaks enriched

|  |  |  |  |  |
| --- | --- | --- | --- | --- |
| **Gene** | **Experiment** | **Number of upstream peaks** | **Enrichment** | **FDR corrected p** |
| ces-1 | CES-1\_Fed-L1-stage-larvae | 26 | 4.88 | 1.6e-10 |
| ces-1 | CES-1\_Larvae-L3-stage | 24 | 4.77 | 2.5e-09 |
| ces-1 | CES-1\_Embryos | 37 | 2.68 | 1.2e-08 |
| ces-1 | CES-1\_Larvae-L4-stage | 21 | 4.18 | 5.5e-07 |
| zag-1 | ZAG-1\_Larvae-L2-stage | 25 | 2.96 | 9.8e-06 |
| zag-1 | ZAG-1\_Fed-L1-stage-larvae | 18 | 3.78 | 3.1e-05 |
| alr-1 | ALR-1\_Larvae-L2-stage | 29 | 2.44 | 3.4e-05 |
| C34F6.9 | C34F6.9\_Larvae-L2-stage | 29 | 2.15 | 4.4e-04 |
| ceh-26 | CEH-26\_Late-Embryonic-stage | 22 | 2.52 | 8.4e-04 |
| fkh-2 | FKH-2\_Larvae-L3-stage | 16 | 3.26 | 8.5e-04 |
| F45C12.2 | F45C12.2\_Fed-L1-stage-larvae | 24 | 2.17 | 3.2e-03 |
| lsy-2 | LSY-2\_Larvae-L1-stage | 30 | 1.86 | 4.2e-03 |
| sem-4 | SEM-4\_Larvae-L2-stage | 16 | 2.77 | 5.2e-03 |
| gei-11 | GEI-11\_Larvae-L2-stage | 21 | 2.21 | 8.1e-03 |
| zag-1 | ZAG-1\_Larvae-L4-stage | 17 | 2.30 | 2.3e-02 |
| fos-1 | FOS-1\_Fed-L1-stage-larvae | 23 | 1.89 | 3.1e-02 |
| gei-11 | GEI-11\_Larvae-L3-stage | 19 | 2.05 | 4.0e-02 |
